# Diagnostics and prospective outcome of a diffuse glioneuronal tumor with oligodendroglioma-like features and nuclear clusters after surgical resection (DGONC): a case report

**DOI:** 10.1093/noajnl/vdac170

**Published:** 2022-10-25

**Authors:** Chelsea Howie, Tahani Ahmad, Kathryn McFadden, Bruce Crooks, P Daniel McNeely, Simon Walling, Robert Rutledge, Felix Sahm, Felix Hinz, Nada Jabado, Mark Kieran, Craig Erker

**Affiliations:** Department of Pediatrics, IWK Health, Halifax, Nova Scotia, Canada; Department of Diagnostic Radiology, IWK Health, Halifax, Nova Scotia, Canada; Department of Pathology, IWK Health, Halifax, Nova Scotia, Canada; Division of Hematology/Oncology, Department of Pediatrics, IWK Health, Halifax, Nova Scotia, Canada; Division of Neurosurgery, Department of Surgery, IWK Health, Halifax, Nova Scotia, Canada; Division of Neurosurgery, Department of Surgery, IWK Health, Halifax, Nova Scotia, Canada; Department of Radiation Oncology, Dalhousie University, Halifax, Nova Scotia, Canada; Department of Neuropathology, Institute of Pathology, Ruprecht-Karls-University, Heidelberg, Germany; Clinical Cooperation Unit Neuropathology, German Cancer Research Center, German Cancer Consortium, Heidelberg, Germany; Hopp Children’s Cancer Center, NCT Heidelberg, Heidelberg, Germany; Department of Neuropathology, Institute of Pathology, Heidelberg University Hospital, Heidelberg, Germany; Clinical Cooperation Unit Neuropathology, German Cancer Research Center (DKFZ), German Consortium for Translational Cancer Research (DKTK), Heidelberg, Germany; Quantitative Life Sciences, McGill University, Montreal, Quebec, Canada; Department of Human Genetics, McGill University, Montreal, Quebec, Canada; Department of Pediatric Oncology, Children’s Cancer Hospital Egypt, Cario, Egypt; Division of Hematology/Oncology, Department of Pediatrics, IWK Health, Halifax, Nova Scotia, Canada

**Keywords:** brain tumor, molecular diagnostics, patient outcomes

Diffuse glioneuronal tumors with oligodendroglioma-like features and nuclear clusters (DGONC) are rare tumors of the central nervous system, having been added as a provisional diagnosis in the 2021 World Health Organization (WHO) Classification of Tumors of the central nervous system (CNS).^1^ Retrospective studies report that these are most often misdiagnosed and treated as high-grade tumors of the CNS.^2,3^ However, this entity has demonstrated superior long-term survival in comparison. Given its novelty, there is currently no standard of care. We describe the diagnostic challenges, molecular characteristics, prospective management, and outcome of a pediatric patient with a DGONC, with the aim to increase awareness of this entity and describe clinical behavior and diagnostic uncertainties.

## Case Presentation

A 6-year-old male presented with a 2-year history of intermittent vomiting that increased in frequency over the preceding 3 months. Additionally, there was an approximate 18-month history of episodes consisting of breath-holding, shaking of hands, vacant staring, and non-sensical speech each lasting about 20–45 s before resolution, and though initially responsive during the episodes, he became progressively less responsive as time went on. The patient presented to their local emergency department after what was determined to be a focal seizure lasting less than 40 seconds. He had 3 to 4 similar seizures over the following 2 days and was started on antiepileptics. Shortly after, he developed an ataxic gait which prompted neuroimaging demonstrating a right hemispheric tumor with midline shift and mass effect.

## Clinical Course

**Figure 1.**
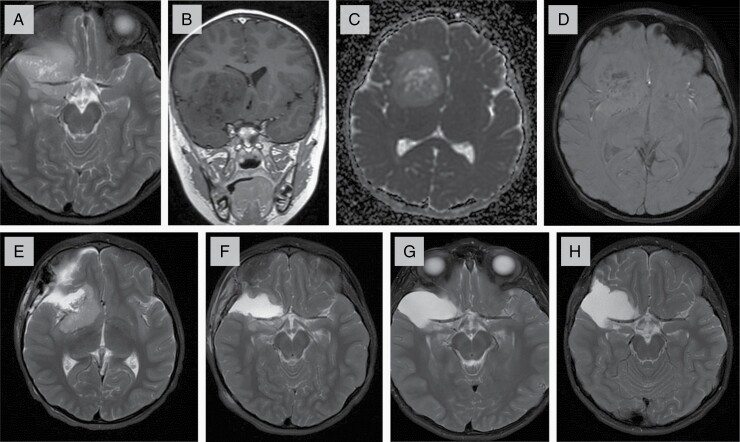
Radiologic features of tumor. (A-D) Axial T2, Coronal T1 post contrast, Axial ADC, and Axial SWAN images of the lesion at presentation. (A) Lesion is well defined, encasing the right MCA. (B) Demonstrates no enhancement post contrast administration. (C) No diffusion restriction noted. (D) Central calcifications seen on susceptibility weighted images. (E-H) Follow-up axial T2 images. (E) Subtotal resection of the tumor after first surgery. (F) Small residual component posterior to the MCA after second surgery. (G) Stable residual lesion on follow-up scans at 3 months, and 1-year post-surgery (H).

Initial MRI showed a large, well-defined intraparenchymal mass lesion involving the right frontal and temporal lobes. There was complete encasement of the right middle cerebral artery (MCA). There was no peritumoral edema, no enhancement, and no restricted diffusion. There were no findings of distant or regional metastases. The MRI features suggested a low-grade lesion (Figure 1a–d).

The patient underwent a frontotemporal craniotomy with a resultant subtotal resection limited due to the MCA (Figure 1e). Postoperatively, he had an uncomplicated recovery.

**Figure 2.**
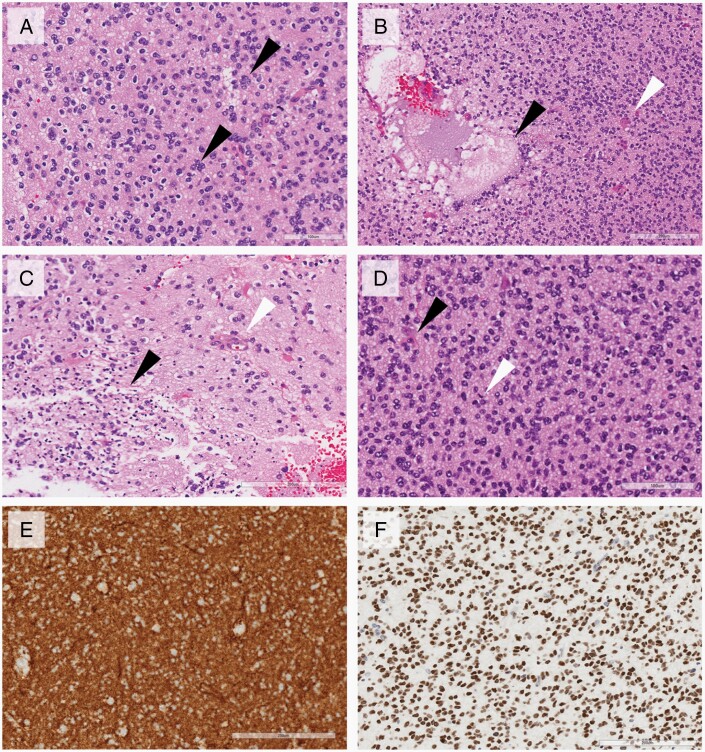
Histopathologic features of tumor. (A) Moderately cellular tumor with round nuclei, perinuclear halos, and nuclear clusters (black arrows). (B) Areas of mucinous degeneration with foam cells (black arrow) and scattered microvascular proliferation (white arrow). (C) Areas of confluent apoptosis (black arrow) and associated endothelial proliferation (white arrow). (D) Frequent entrapped normal neurons (black arrow) and mitotic figures (white arrow). (E) Strong diffuse positivity for synaptophysin. (F) Strong diffuse nuclear staining for OLIG2. Scale bar represents 200 microns.

The histopathology of the initial surgical resection was initially thought to be consistent with a high-grade glioma. Histology sections showed a moderately cellular, diffusely infiltrating tumor composed of round cells with variable perinuclear halos (Figure 2a). Scattered nuclear clusters were evident. Areas of myxoid degeneration with foam cells and early cystic change were also noted (Figure 2b). Mitotic figures and single cell necrosis were frequent and occasional microvascular proliferation and confluent necrosis were present (Figure 2c and d). The tumor cells were negative for GFAP which only highlighted reactive background astrocytes. Neuronal markers, such as synaptophysin and NeuN, were strongly and diffusely positive, as was OLIG2 (Figure 2e and f). Immunohistochemistry for P53 showed a wildtype pattern, and *BRAF* V600E and IDH1 R132 were negative. Mismatch repair proteins were retained. The Ki67 proliferation rate approached 25% focally.

**Figure 3.**
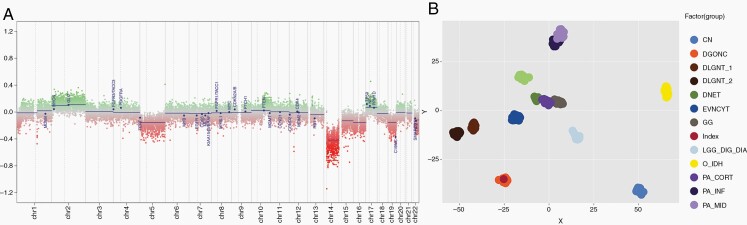
Molecular features of tumor. (A) Copy number variation plot showing the characteristic loss of chromosome 14. (B) tSNE embedding of the index case in regard to selected tumor types central neuroblastoma (CN), diffuse glioneuronal tumors with oligodendroglioma-like features and nuclear clusters (DGONC), Diffuse leptomeningeal glioneuronal tumor subgroup 1 and 2 (DLGNT_1 and _2), dysembryoplastic neuroepithelial tumor (DNET), extraventricular neurocytoma (EVNCYT), ganglioglioma (GG), desmoplastic infantile ganglioglioma/astrocytoma (LGG_DIG_DIA), oligodendroglioma (O_IDH), pilocytic astrocytoma cortical, infratentorial and midline (PA_Cort, _INF & _mid), rosette-forming glioneuronal tumor (RGNT).

Whole chromosomal aberrations showed monosomy 14, mosaic loss of one copy of chromosomes 5, 15, 16, 19, and 22, gain of one copy of chromosomes 2 and 17, and mosaic copy neutral loss of heterozygosity of chromosome 13 (Figure 3a). There was no *1p/19q* co-deletion and no *7q34* duplication with breakpoints in *KIAA1549* and *BRAF*. Additional molecular analysis, including a RNA-derived NGS panel as well as whole genome and RNA sequencing, showed no known pathogenic rearrangements. It was subsequently sent for a DNA methylation array at Universitats Klinikum Heidelberg in Germany. DNA methylation using the brain tumor classifier version 12.5 returned showing a calibrated score of 0.93 for a DGONC (Figure 3b).

It took approximately 3 months to obtain a clear and accurate diagnosis due to challenges posed by this rare tumor entity including some high-grade features on histopathology (Supplementary Figure 1). The diagnostic difficulty was compounded by the lack of routine methylation classification at our centre. During this time of securing a diagnosis, the patient did well and remained seizure free. Given the findings which were initially concerning of a high-grade glioma, maximal resection was pursued and an MRI prior to this repeat surgery, 3 months since previous imaging, showed that the residual tumor had not grown on repeat MRI. The patient underwent an image guided right frontotemporal craniotomy and duraplasty. Resection remained subtotal again due to challenges presented by MCA encasement (Figure 1f). Given the stability of the tumor over the initial 3 months between the 2 surgical resections (Figure 1g), the prolonged symptom interval, and that no standard of care is known for DGONC, a watch and wait approach without adjuvant therapy was undertaken. The patient’s residual tumor has since been followed and remains stable without growth 14 months from the second surgery and 17 months from original diagnosis (Figure 1h). The patient continues to be clinically well and is thriving.

## Discussion

The literature on DGONC as a unique entity is sparse, with it being named as one of the 3 provisional entities in the 2021 WHO Classification of Tumors of the CNS.^1^ DNA methylation is a useful and precise method of classification of CNS tumors that was very helpful in securing the diagnosis for our patient. It has led to the identification of new, molecularly defined CNS tumor types and subtypes and has been shown to lead to a change in diagnosis in up to 12% of prospective cases.^4^

This entity was initially described by Deng et al. in 2020 who screened genome-wide DNA methylation data of over 25,000 CNS tumors for clusters of tumors separated from other defined DNA methylation classes. They demonstrated a novel group of 31 tumors termed as DGONC. The original histological diagnoses were available for 29 of the 31 tumors and included entities such as primitive neuroectodermal tumors (31%), atypical extraventricular neurocytoma (17%), glioblastoma (14%), anaplastic oligodendroglioma (14%), and low-grade glioma (10%).^2^ Subsequently, a case series by Pickles et al. reviewed 123 CNS tumor cases with undetermined final diagnoses using DNA methylation and those that had monosomy of chromosome 14. They found 3 tumors with similar methylation profiles to those described by Deng et al, with prior diagnoses of either malignant glioneuronal tumor or high-grade neuroepithelial tumor.^3^

In terms of histology, DGONCs have been described as having a clear cell appearance, perinuclear haloes, vascular proliferation, and nuclear clusters.^2,3^ Calcification, ganglion cells, apoptosis, and foamy cells have also been seen.^3^ Strong MAP2, NeuN, OLIG2, and synaptophysin positivity with predominant GFAP negativity has been noted.^2,3^ The mitotic index has ranged from 0.42 to 3.38 per mm^2^, and focal positivity for Ki67 of up to 30% have been observed in prior studies.^3^ Our case exhibited many of the same features worrisome for aggressive behavior commonly observed in other diffusely infiltrating gliomas. The focal Ki67 rate of up to 30% along with a mitotic count of approximately 9 per 10 high power fields, generated significant discussion about the need for aggressive therapy in the context of a high-grade lesion. Single-cell and confluent apoptosis and microvascular proliferation were also frequent. In retrospect, however, these findings were more evident adjacent to areas of mucinous degeneration and thus may reflect that process rather than an increased tumor grade.

Deng et al. described monosomy of chromosome 14 as a uniting feature in 30 of 31 tumors; other genetic findings included gain of 17q (58%) and 1q (26%), and loss of 19q (35%).^2^ In the 3 cases described by Pickles et al., monosomy of chromosome 14 was found in addition to a common focal loss of chromosome 1p and loss of chromosome 3p on the copy number plots.^3^ Our case also demonstrated a loss of chromosome 14 in addition to a gain of chromosome 17 and a mosaic loss of chromosome 19, with no *BRAF* alterations, thus showing similar cytogenetic changes to previously described DGONCs. Few studies have shown the presence of tumor suppressor genes located on chromosome 14q.^5,6^ Though the loss of such genes may be involved in the pathogenesis of DGONCs, we cannot confirm this based on our case and future research is needed.

Radiologic features of DGONC are not well described. Deng et al. described their tumors as being within the cerebral hemispheres, stemming mainly from the temporal lobes, and did not exhibit tumor dissemination at first presentation.^3^ The tumors studied by Pickles et al. were well-circumscribed cortical or subcortical supratentorial masses with no lobar predilection.^3^ Each of these 3 tumors had no perilesional edema, were hyperintense compared to the cortex on T2 and FLAIR weighted sequences and were poorly enhanced with contrast. The tumors also showed calcification of the internal matrix, with a heterogenous signal on diffusion-weighted images and a small focus of low central apparent diffusion coefficient (ADC)^3^ Our patient’s tumor similarly demonstrated no surrounding edema, heterogenous low T1 and high T2 and FLAIR signal intensity, no diffusion restriction, and no significant post-contrast enhancement. This tumor was predominately solid with multiple small cystic spaces and coarse central calcifications. The lack of perilesional edema, and no diffusion restriction at presentation is unusual for most high-grade tumors, which made our team question the concerning high-grade features noted above and consider a low-grade lesion. The lack of growth between the 2 surgeries further supported this impression.

Clinically, Deng et al. found that patients with DGONC had a median age of 9 years with no sex predilection. Though many initial histological diagnoses were high-grade, available follow-up data showed a favorable clinical course with disease progression in only 3 patients. With 2 of these patients had delayed mortality at 25 and 96 months which suggests prolonged follow-up should be considered. However, no therapeutic data were discussed, and availability of follow-up data was limited.^2^ All 3 patients described by Pickles et al. are alive and had favorable outcomes; all patients underwent total gross resection and craniospinal radiation in addition to chemotherapy and are considered to be in complete remission.^3^ Despite these positive outcomes, we recognize the overall lack of treatment and outcome data for this population, making it difficult to draw definitive conclusions on prognosis. Though our patient is younger in age than those previously described, he has done exceptionally well through close follow-up. He has not undergone any chemotherapy or radiotherapy, and though there is residual tumor, this has exhibited no growth over the 17 months since initial diagnosis.

Our case was unusual as the symptom interval was prolonged and imaging features were not consistent with a typical high-grade glioma, presenting a diagnostic dilemma that was clarified with the assistance of DNA methylation. Symptom interval for DGONC has not been previously discussed, and there are no studies discussing the optimal treatment of DGONCs. This is the first case in the literature to date that demonstrates prospective follow-up of a DGONC after subtotal surgical resection with no adjuvant therapies; our patient has done well without progression of residual disease. Given the current literature as well as our findings, DGONC is a rare entity that is frequently misdiagnosed and potentially over-treated. At present, classic histopathology and imaging features along with the presence of monosomy 14 strongly suggest a diagnosis of DGONC, especially in cases where methylation profiling is not readily available. The clinical course of our case is encouraging and while wide applicability needs to be cautioned with a single case, close observation following surgical resection of DGONCs may be a consideration in the context of multidisciplinary team review with the possibility to reduce short- and long-term complications associated with chemotherapy and radiotherapy.

## Supplementary Material

Supplementary material is available online at *Neuro-Oncology Advances* online.

Supplementary Figure 1. Timeline of diagnostic events. Magnetic Resonance Imaging (MRI), subtotal resection (STR). Immunohistochemistry (IHC), single-nucleotide polymorphism (SNP), Diffuse Glioneuronal Tumor with Oligodendroglioma-like features and Nuclear Clusters (DGONC)

vdac170_suppl_Supplementary_Figure_S1
