# Supplementary figures and images for: Diagnostics and prospective outcome of a diffuse glioneuronal tumor with oligodendroglioma-like features and nuclear clusters after surgical resection (DGONC): a case report
Source: Neurooncol Adv. 2022 Oct 25;4(1):vdac170. doi: 10.1093/noajnl/vdac170 (PMC9719366; doi:10.1093/noajnl/vdac170)

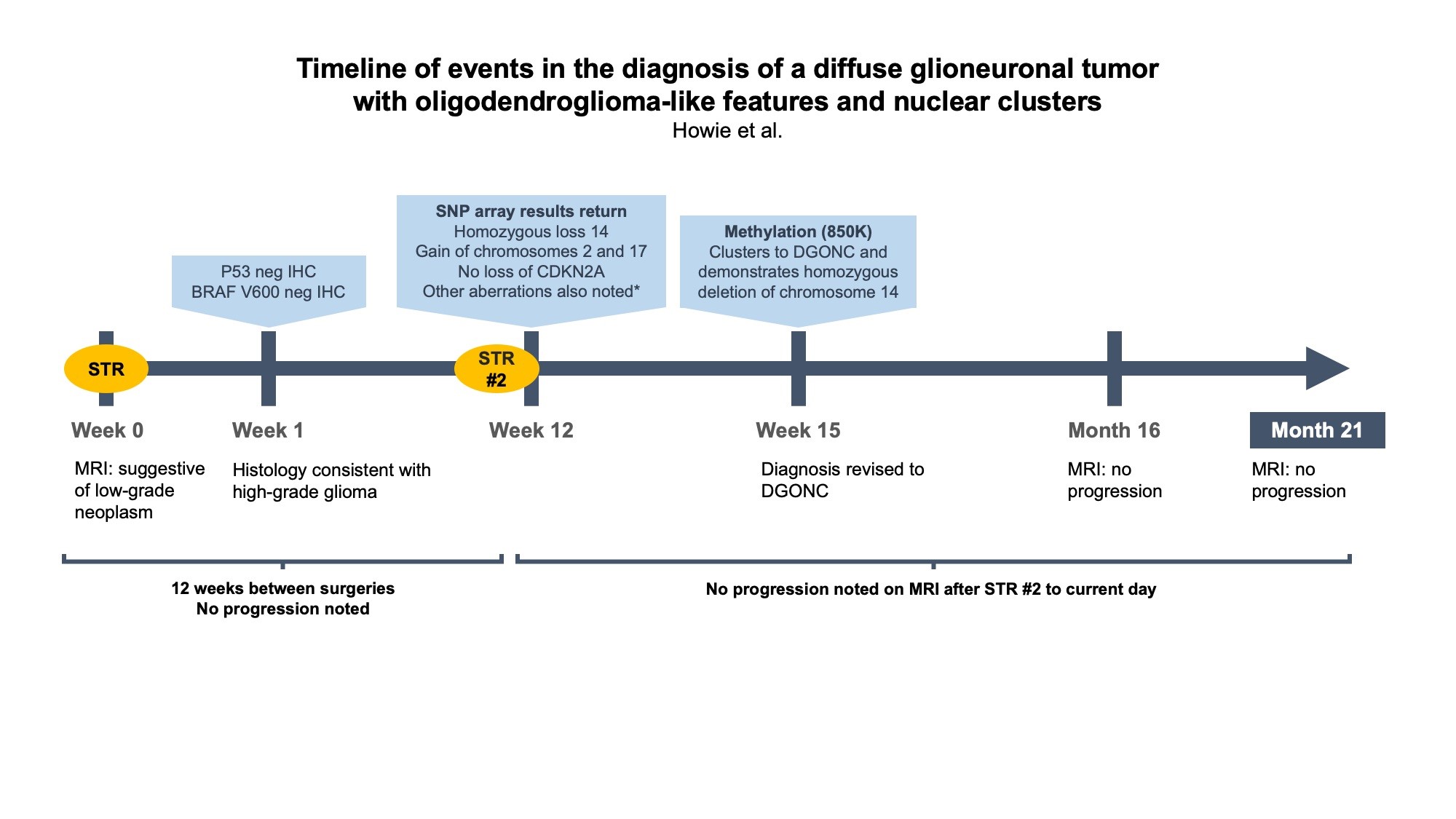

Supplement: vdac170_suppl_Supplementary_Figure_S1 [file vdac170_suppl_supplementary_figure_s1.jpeg]
